# The Dignity of Medicine

**Published:** 1888-01-28

**Authors:** 


### The Hospital

A Weekly Institutional Journal of

Science, Medicine, and Philanthropy.

*For Hospitals, Asylums, Medical Practitioners, Students, Nurses, Families, Parishes, Congregations, and the Charitable Public.*

Vol. III. No. 70.] [January 28, 1888.

### The Dignity of Medicine.

Every calling in organised society has a place and rank of its own, a rank acquired and maintained by the action of all those forces which promote social development. It is instructive to note that precedence is not given to callings which are most directly useful to man, and on which he is immediately dependent, but to those which tend to raise the character of their professors to the highest intellectual and moral level. The agricultural labourer is the man of all others who is absolutely indispensable to the existence of himself and his fellows. Without him, corn and cattle, bread and meat would fail, and a destructive famine would immediately result. On the other hand, the religious teacher exercises a function which in no way concerns the mere animal life. So far as bodily existence and health are concerned nobody need be any the worse if every religious teacher were silenced at once and for ever. But in spite of these obvious and universally-admitted facts the world—not the Church—but the huge, common-place, eating and drinking, everyday world, places the religious teacher by common consent at the head of the active professions, and the agricultural labourer very near the foot. This is a practical judgment of human affairs worth thinking about.

The medical profession has its recognised and definite place in the social organism. That relatively high position results, not from the peculiar service which the professors of medicine render to mankind, but from the fact that medical men engage in liberal studies which tend to raise their intellectual and moral level. This is well seen by reference to the history of medical progress even within such a comparatively brief period as the present century. Many of the older physicians and surgeons can well remember the equivocal position occupied by the average practitioner of medicine in their boyhood as compared with that to which he has attained at the present time. To-day the doctor is *ipso facto* a man of education and breeding. If in exceptional instances he cannot be so described, it is recognised that the instances are exceptional, and that these cases do not invalidate the rule any more than similar cases among the clergy or any other body of men of admitted status and culture.

Medicine is one of the "learned professions." The meaning of this is that its followers are persons who have received a liberal education. But what is the object of a liberal education? Is it merely to make men acquainted with a certain number of matters of fact or of mathematical or grammatical rules? The object of the preliminary examination in arts to which men are subjected before entering upon their special studies is to ensure that none but those who have mastered at least the rudiments of a liberal education shall be allowed to pass the portals of medicine; and the object of a liberal education is to develop the mind into a seeing, reasoning, and discriminating instrument, as opposed to a stupid, impulsive, prejudiced, and unreasoning faculty. The ignorant person has not the power either to see or to understand unfamiliar things. Difficult problems in logic, morals, or science are not for him. He goes off at a tangent, acts upon an impulse, fails to distinguish between expediency and principle, calls great things small and small things great, has no intelligent persistence, no moral and intellectual steadiness, strength, and dignity. It is, or ought to be, otherwise with the man of education. He is, or ought to be, able to weigh and to measure, to take the true and proper bearings of a subject; to deal with ethical, moral, and scientific questions in a broad and judicial spirit; to distinguish between the accidental and the essential, the expedient and the fundamental, in every case in which he is called upon to form a judgment or to deliver a verdict. The ability and the resolution to do these things constitute the true dignity of the learned professions, as they constitute the true dignity and value of man as an intellectual being. Any creature, the lowest animal, can act from instinct and impulse; any man, the most stupid and vicious, can act from self-interest and prejudice. But to act with dignity is to act with intelligence, with self-repression, with discrimination, and according to the highest standards of moral rectitude and impartial justice; and this can be done by cultivated intelligence alone. Any departure from these on the part of an intellectual being like man is not dignity, but degradation, however loftily it may be done as to manner, and however it may be applauded by the shallow, the cynical, and the self-seeking.

What is dignity? It seems childish to ask the question; and we would not ask it were it not that so many mistake the manner for the matter, the husk for the kernel. Two colleagues in a certain medical practice visited alternately a lady of the old school, since dead, during her last illness. She was a strong Conservative and a firm believer in old-fashioned courtesies and demeanour. "I like Dr. —— well enough," she said to a friend, "and he agrees with me in politics; but he has a *bedside manner*, and you feel you are not dealing with the real man—you are not certain of him. Now, Dr. ——," naming the other, "comes into the room with a frank and easy simplicity, as if he were not entering a sick room at all. When he sits down by the bedside, and begins his questions and comments, you feel that he is treating you as he would like to be treated himself if he were the patient. He talks to you reasonably and as if he were talking about any other business in which he was earnestly concerned; and when he has gone away you are conscious that somebody has been with you who knows quite well what you think and feel, and what your condition is, and who will do you good if he can." This episode answers the question about the nature of dignity. Dignity is not pomp, and affectation, and frock-coats, and well-pared nails. It is worth, simplicity, kindness, and, as Mr. Matthew Arnold would say, "sweet reasonableness."

Why do so many members of the medical profession excel in dignity, as it is admitted that in many departments of social life they undoubtedly do? Because their calling is such that they are in the daily practice of patience, gentleness of manner, sympathy with suffering, and even with wilfulness. Of many individual medical men it may be said with truth that they attain to the highest excellence both in manners and character. Such men feel that they owe it to themselves always to think, feel, and behave with that true and real dignity which is of the very essence of the purest moral worth.

*Noblesse oblige.* That which a man owes to himself he owes quite as much to others, and, if possible, in an especial degree to other individual members and to the whole body of his own profession. As he would not be prejudiced and illiberal at the bedside of his patient, so he must not, when associated with his fellows in a corporate capacity, permit himself or permit them so to be if he can help it. The honour of a great profession is in his hands, and the amount of that can never be greater than that of all the individuals of which the profession is composed. Medicine is naturally liberal to overflowing; and just as a true physician when in the sick-room feels that there is nothing he would not do or deny himself if thereby he could advantage or save the life of his patient, so, when acting with the whole body of the profession, should he feel that the whole art and science of medicine are for the health and well-being of all mankind, and that the interests of the world must take precedence of the interests of a limited class. He must and will act so that men shall be encouraged in every way to avail themselves intelligently of the progress of medicine as well for the preservation as for the restoration of health. The mean and narrow-souled journeyman who demands his sixpence per hour, and would not work a minute beyond the hour without his fraction of sixpence to save his master from bankruptcy, is not the type of man who should belong to any learned profession; least of all to that which concerns itself with such primary and fundamental interests as health and life. The true dignity of the whole profession of medicine, as of each individual member of it, consists in its intrinsic worth, its broad and deep intelligence, its liberality of soul, its generosity of spirit, its earnest desire and high resolve to build up and make available for all men a true science of health and a competent art of healing. Aims like these ennoble both men and their arts; and it may be hoped for ours as for other professions that no lower will ever find place among those who bear the banner and march in the foremost ranks.

